# Imaging Findings of Ovarian Vein Thrombosis

**DOI:** 10.7759/cureus.48672

**Published:** 2023-11-11

**Authors:** Mohamed T El-Diasty, Yasser Noorelahi

**Affiliations:** 1 Radiology, King Abdulaziz University Hospital, Jeddah, SAU; 2 Radiology, King Abdulaziz University Faculty of Medicine, Jeddah, SAU

**Keywords:** mri-magnetic resonance imaging, computed tomography (ct ), post partum, thrombosis, idiopathic ovarian vein thrombosis

## Abstract

Ovarian vein thrombosis (OVT) is an uncommon condition that occurs mainly in the peripartum period. Hyper-coagulable conditions have been reported to cause OVT outside the peripartum period. The clinical presentation is usually nonspecific pain, but it can be asymptomatic in patients with underlying malignancy. Imaging plays an important role in diagnosis. Ultrasound is the initial imaging modality, but it is operator-dependent and has limited sensitivity. Computed tomography (CT) is the most commonly used modality for diagnosis. CT can show the luminal filling defect within the thrombosed vein and assess the extension of the thrombosis. MRI can show the thrombosed vein as a filling defect on post-contrast images; also, diffusion-weighted images may help in the diagnosis. Complications include extension into the inferior vena cava or renal veins. Pulmonary embolism is the most serious complication. Treatment includes anticoagulation plus antibiotics. Early diagnosis is essential to prevent complications.

## Introduction and background

Ovarian vein thrombosis (OVT) is an uncommon condition that commonly affects women in the partum and peripartum periods. The incidence of OVT detection has increased due to increased utilization of imaging studies. Conditions that increase blood coagulability, such as malignancy, surgery, trauma, infection, recent hospitalization, or hereditary thrombophilia have been reported to cause OVT outside the peripartum period. When OVT occurs without predisposing factors, it is referred to as idiopathic ovarian vein thrombosis [1,2].

## Review

Ovarian veins anatomy

**Figure 1:**
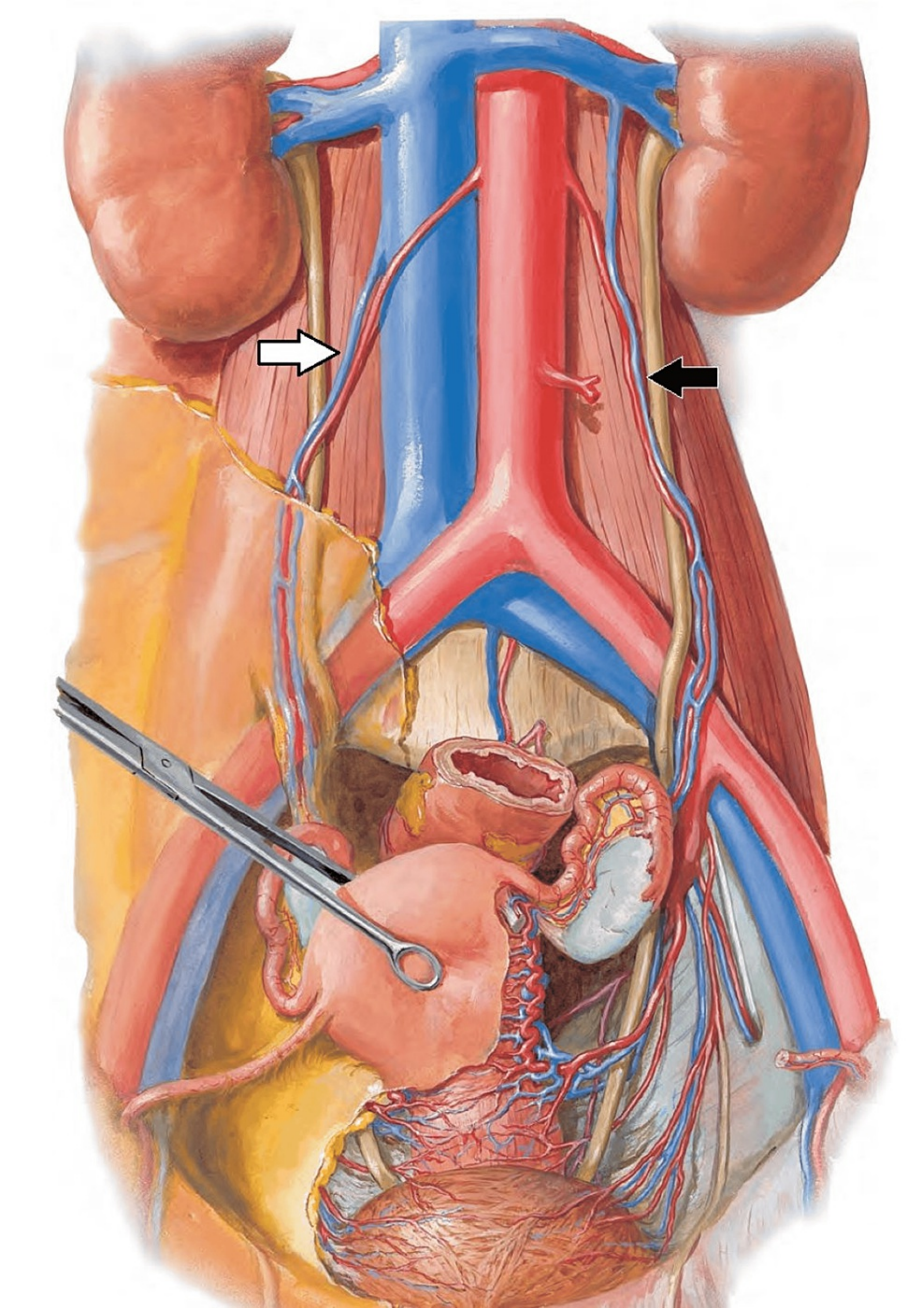
Ovarian veins anatomy The illustration demonstrates ovarian veins arising from the ovarian venous plexus on both sides. The white arrow shows the ovarian vein draining to the inferior vena cava (IVC) on the right side, while on the left side, the ovarian vein (black arrow) drainage is through the left renal vein Image source: Atlas of Human Anatomy 6th Edition, Frank Netter, with permission

The origin of ovarian veins is the broad ligament venous plexus, which communicates with the uterine plexus, afterwards, the ovarian vein passes superiorly along and anterior to the psoas muscle. The drainage of the right ovarian vein is commonly to the inferior vena cava (IVC), while the left ovarian vein drains into the left renal vein (Figure 1) [3].

On cross-sectional imaging, the ovarian veins are best visualized at the level of the inferior mesenteric artery origin, where both veins are surrounded by retroperitoneal fat [4].

Pathophysiology of ovarian vein thrombosis

**Figure 2:**
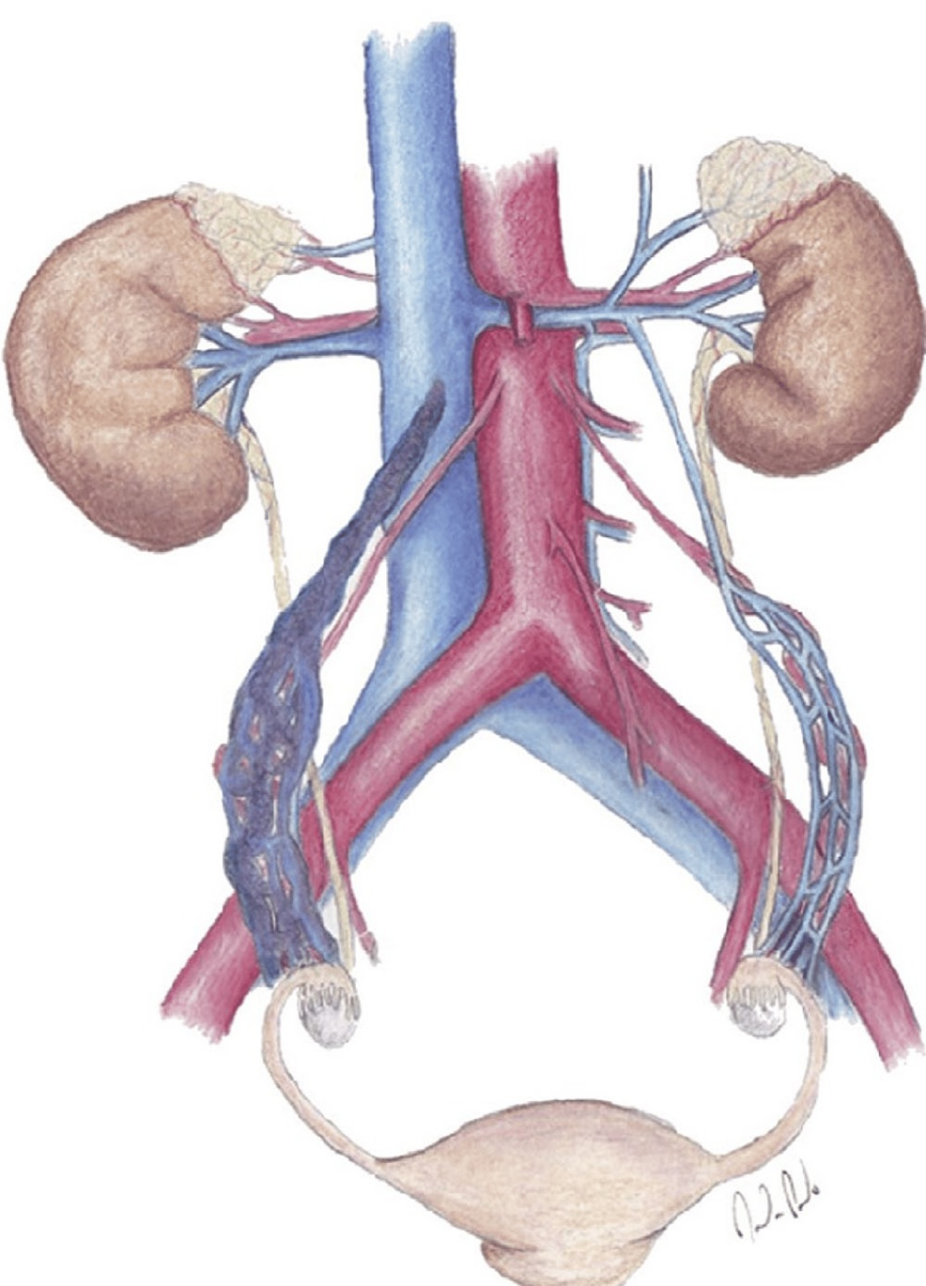
Illustration demonstrates thrombus propagation from the ovarian venous plexus into the right ovarian vein From reference [5], with permission.

Pregnancy-related OVT is caused by the hormonal changes causing blood hypercoagulability, in addition, venous stasis from compression of the growing uterus and possible endothelial injury during delivery, all these factors complete the Virchow’s triad of venous thrombosis. Thrombus develops in the ovarian plexus and then propagates through the ovarian vein (Figure 2) [5].

In individuals outside the peripartum period, thrombophilia disorders, malignancy, and infection are contributing factors in developing such conditions [6]. Coronavirus disease 2019 (COVID-19) is well known to cause a general hypercoagulable state and has been associated with many cases of stroke, myocardial infarction, and deep venous thrombosis. OVT was also reported with COVID-19 infection in a few case reports [7].

Clinical features

The incidence of postpartum OVT ranges from one in 600-7000 deliveries, it is also suggested that the incidence is much higher due to the nonspecific symptoms. The post-partum patients usually present within two weeks after delivery with lower abdominal pain, fever, and leukocytosis. When OVT occurs on the right side, patients may present with abdominal pain which simulates acute appendicitis [6]. OVT develops frequently in post-hysterectomy and/or oophorectomy patients; also, it has been reported to occur in patients with malignancy [8]. When patients are outside the peripartum period, the diagnosis is challenging and requires a high suspicion index. OVT can also occur after abdominal surgeries rather than hysterectomy [9]. Many conditions can simulate OVT and thus should be included in the differential diagnosis, these include pelvic inflammatory disease, endometritis, pyelonephritis, nephrolithiasis, acute appendicitis, and ovarian torsion [5,6].

Imaging features

Contrast-enhanced CT is the modality of choice for diagnosing OVT. Ultrasound and MRI have also been used for diagnosis.

1) Ultrasound (US)

**Figure 3:**
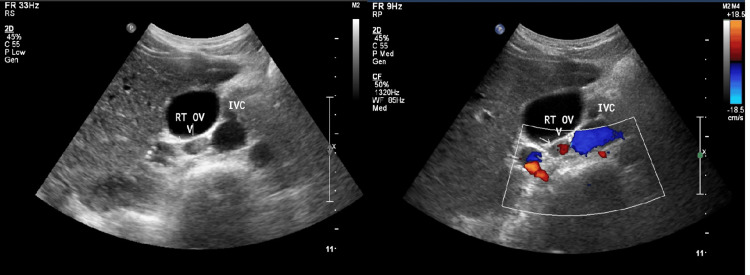
B mode and color Doppler ultrasound images The images demonstrate an echogenic thrombus within the right ovarian vein, reaching but not extending inside the inferior vena cava (IVC).

It is usually the first line of investigation. When OVT is present, the vein usually enlarges with an intra-luminal echogenic thrombus. Color Doppler demonstrates absent, decreased, or marginal flow (Figure 3). The disadvantages of the US are decreased sensitivity compared to CT and MRI especially in obese patients; also it is operator dependent which leads to false negative results [10,11].

2) Computed Tomography (CT)

**Figure 4:**
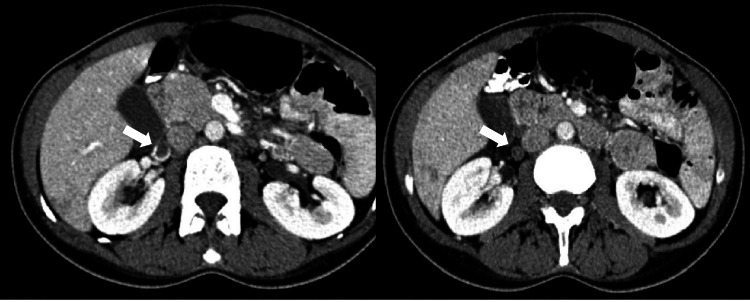
Contrast-enhanced CT (axial) Axial post-contrast CT images at two different levels in a 39-year-old female patient outside the peripartum period demonstrate a distended right ovarian vein (white arrows) with a luminal filling defect and marginal blood flow. This patient was treated by the authors at King Abdulaziz University Hospital.

**Figure 5:**
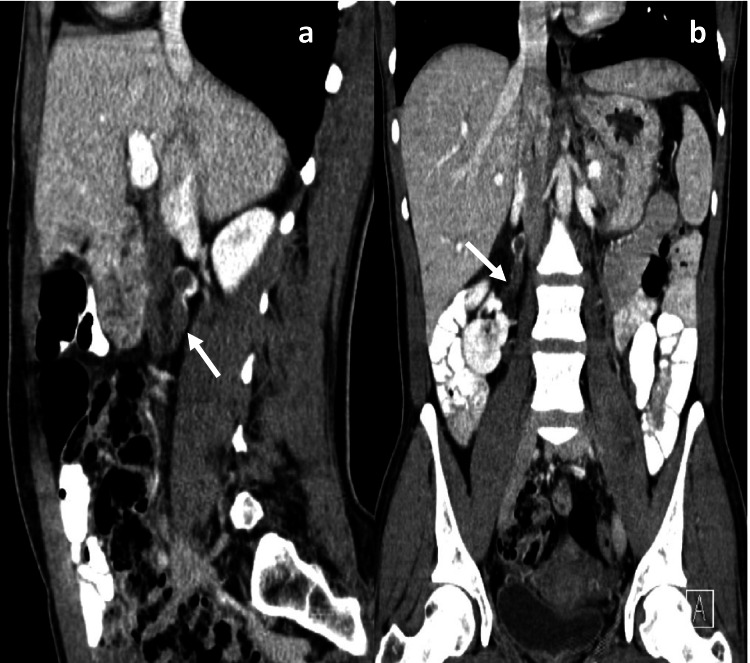
Post-contrast CT images Sagittal (a) and coronal reformatted (b) post-contrast CT images of the same patient demonstrate the superior extent of the thrombus into the upper part of the right ovarian vein (white arrows). This patient was treated by the authors at King Abdulaziz University Hospital.

**Figure 6:**
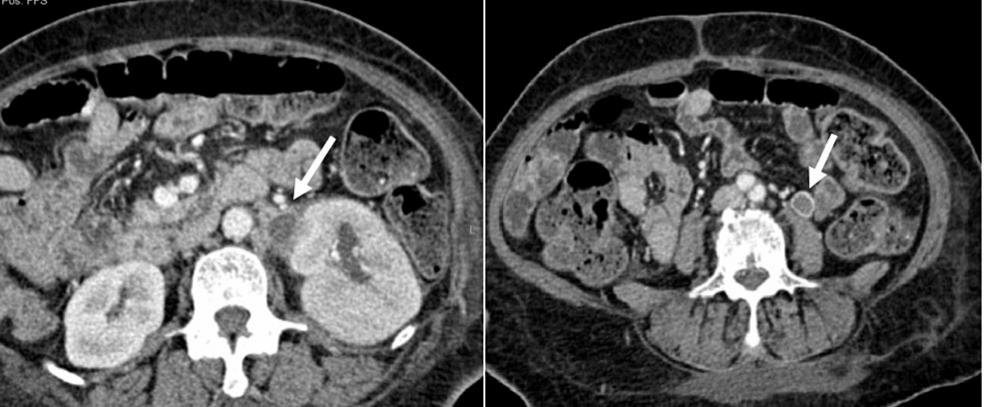
Post-contrast CT images (axial) Two axial post-contrast CT images demonstrate a thrombosed left ovarian vein (white arrows) due to left pyelonephritis in a 41-year-old diabetic female patient treated by the authors at King Abdulaziz University Hospital.

**Figure 7:**
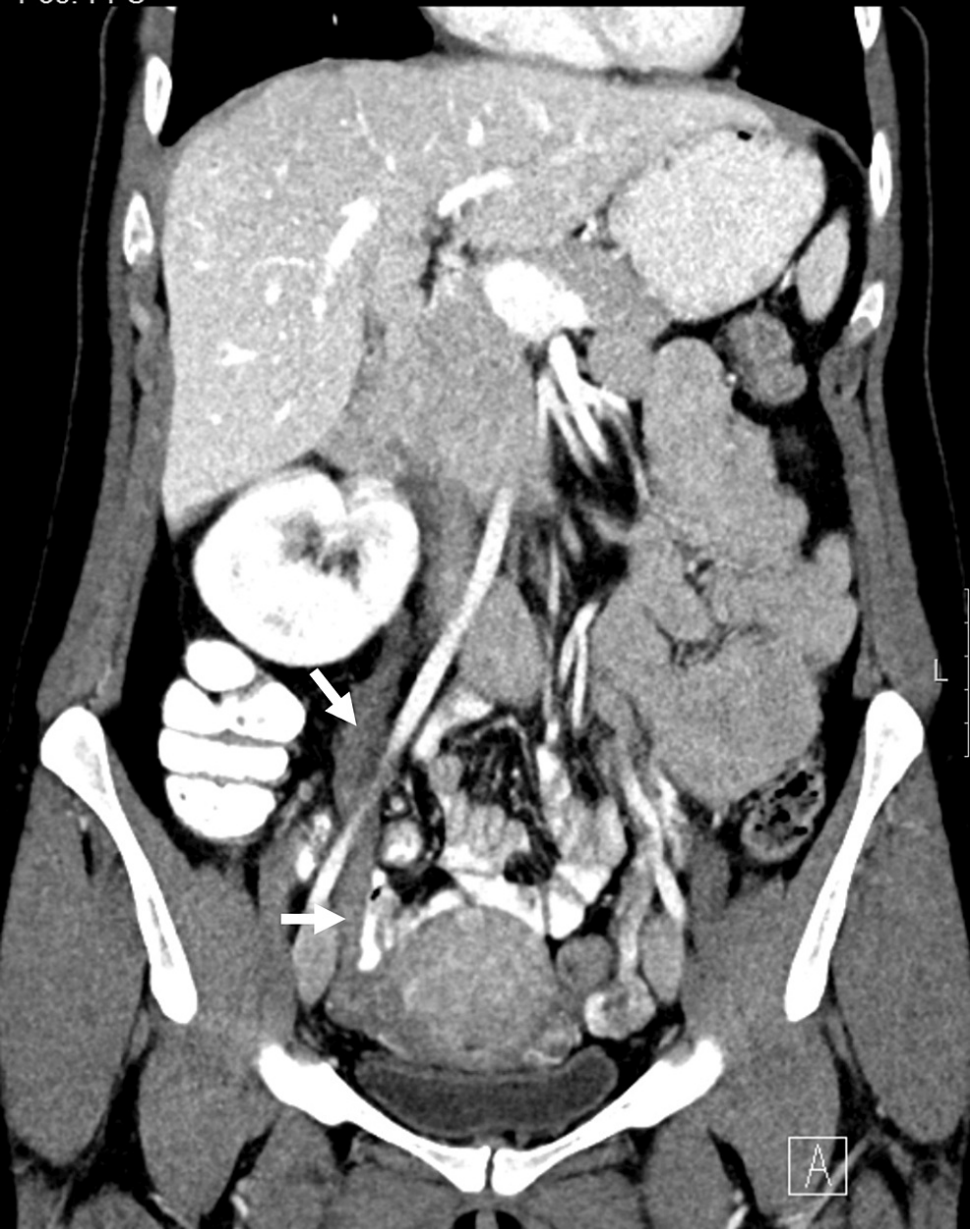
Post-contrast CT image (coronal) Coronal reformatted post-contrast CT image demonstrates distended thrombosed right ovarian vein (white arrows) in a 32-year-old female at day 6 post-cesarean section. This patient was treated by the authors at King Abdulaziz University Hospital.

Image acquisition should be performed in the porto-venous phase for optimal contrast enhancement of the ovarian veins. The timing of the venous phase is usually between 60-80 seconds after contrast administration. CT allows for the detection of thrombus extension into the renal veins and IVC. Assessment of thrombus extension may be limited by the contrast mixing artifact [10]. Classically, OVT appears as a filling defect within a distended ovarian vein on contrast-enhanced CT (Figures 4-7).

OVT can be an incidental finding in patients with malignancy, and it has been reported incidentally on fluorodeoxyglucose positron emission tomography/computed tomography PET-CT in a patient with recurrent carcinosarcoma of the uterus, in such cases, the OVT was due to malignant thrombus extension into the ovarian vein [12].

3) Magnetic Resonance Imaging (MRI)

**Figure 8:**
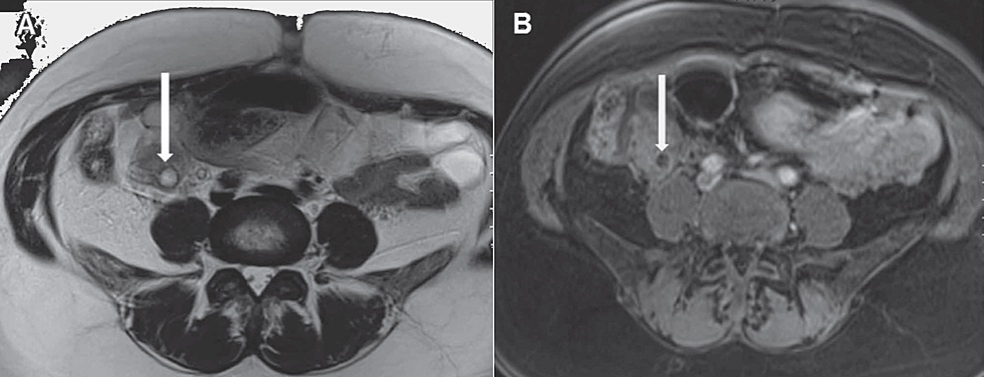
T1 and T2 weighted images (axial) (A) Axial T2-weighted image shows a rounded hyperintense structure (arrow) in the center of an ill-defined mass located at the ventral aspect of the right psoas muscle corresponds to thrombus formation within the right ovarian vein (B) Axial post-contrast T1 weighted image shows the lack of enhancement in the right ovarian vein (white arrow) due to thrombosis. From reference [13], with permission.

**Figure 9:**
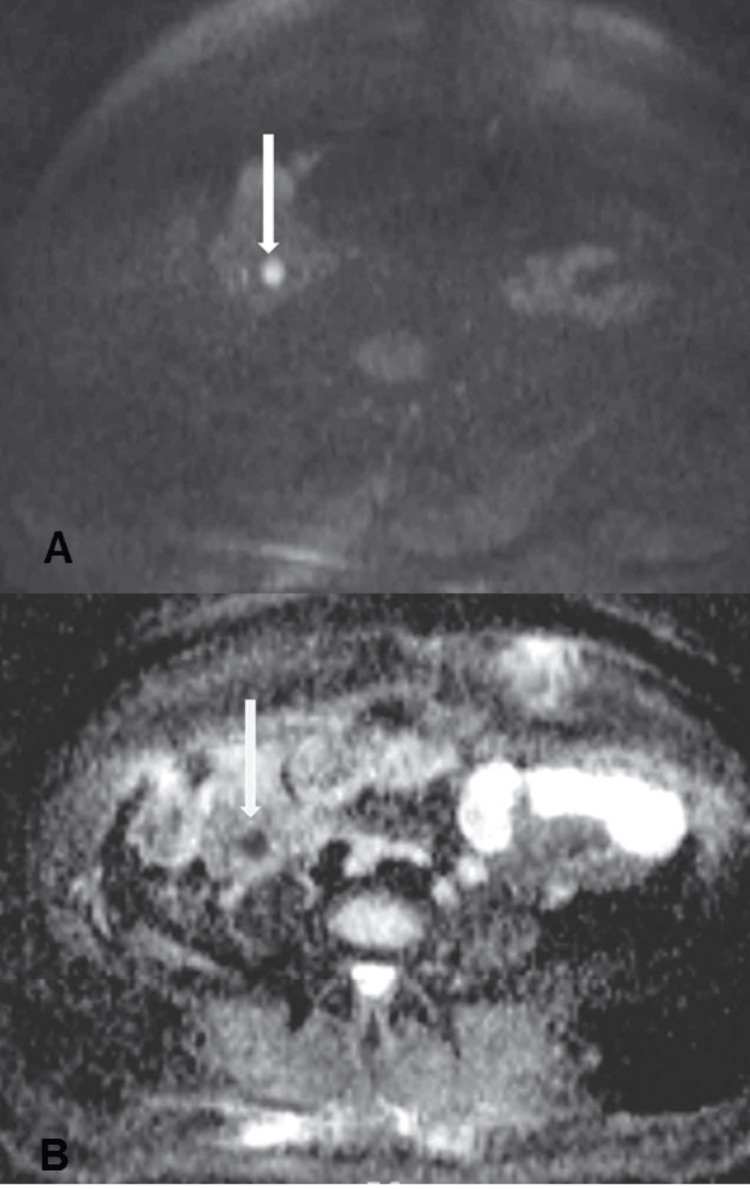
Axial diffusion-weighted image (b value = 800) (A) shows a focal area of hyperintensity (white arrow), and the corresponding ADC map (B) shows a hypointense signal along the course of the right ovarian vein (white arrow). From reference [13], with permission.

The MRI protocol usually includes axial T1 & T2 weighted sequences with the administration of intravenous gadolinium. Magnetic resonance venography (MRV) can be done without contrast using the two-dimensional time-of-flight (TOF) technique. Another technique is the contrast-enhanced MRV utilizing three-dimensional spoiled gradient recalled echo (3D SPGR) acquisition [10]. The appearance of thrombosis on MRI is a hypointense filling defect on contrast-enhanced T1-weighted sequences, while it is usually hyperintense on T2-weighted images. Diffusion-weighted imaging (DWI) is not usually required for diagnosis, however, when used, the acute thrombus will show restricted diffusion (Figures 8, 9) [13].

Prognosis and treatment outcome

The prognosis is usually good; however, complications can occur including sepsis and thrombus propagation to the IVC or renal veins. When IVC thrombosis occurs, the risk of pulmonary embolism is high with an incidence of about 13% and a 4% risk of mortality [14].

The treatment of OVT is controversial. In peripartum ovarian vein thrombosis, intravenous anticoagulation, intravenous antibiotics, or both are given on an inpatient basis followed by three to six months of outpatient oral anticoagulation. In patients with concern for infection, a one-week course of oral antibiotics has also been recommended. To date, there is no consensus regarding the standard duration of anticoagulation or antibiotics. A 2-3-week course of therapeutic anticoagulation is usually used for the treatment of small pelvic vein thrombosis. Longer anticoagulation is recommended for three to six months if the ovarian vein is involved. When renal veins or IVC are involved, the treatment is the same as pulmonary embolism. It is thought that OVT in patients with underlying malignancy usually resolves spontaneously; thus anticoagulation is not required. For post-partum patients with isolated OVT, thrombophilia workup is not indicated. In cases of IVC extension, placement of IVC filters may be indicated to decrease the risk of PE [5,10].

## Conclusions

OVT is an important cause of pelvic pain, especially in peripartum patients. Radiologists should exclude OVT as a cause of acute abdomen especially in women outside the peripartum period. Imaging of OVT includes ultrasound as a primary imaging modality. Contrast-enhanced CT or MRI is usually performed to confirm the diagnosis and exclude other differentials. Complications of OVT are thrombus propagation and pulmonary embolism. Early treatment of OVT is essential to prevent complications.
